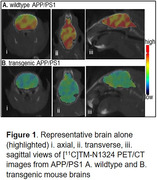# First GPR39‐imaging radiotracer to track zinc homeostasis in a rodent model of Alzheimer’s disease

**DOI:** 10.1002/alz.089711

**Published:** 2025-01-09

**Authors:** Krishna Kumar Gollepalli, Bhuvanachandra Bhoopal, Naresh Damuka, Ivan Krizan, Mack Miller, JV Shanmukha Kumar, Nagaraju Bashetti, Christopher T Whitlow, Samuel N. Lockhart, Pooja Jadiya, Kiran K. Solingapuram Sai

**Affiliations:** ^1^ Wake Forest University School of Medicine, Winston‐Salem, NC USA; ^2^ Koneru Lakshmaiah University, Guntur, AP India

## Abstract

**Background:**

The G protein‐coupled receptor GPR39 is heavily associated with the pathogenesis of neurologic disorders, including Alzheimer’s disease (AD) and related dementia (ADRD). Its dysregulation of zinc 2+ (Zn^2+^) processes triggers metallic dyshomeostasis, oxidative stress, neuroinflammation, microtubule destabilization, synaptic dysfunction, and tau phosphorylation—all hallmarks of neurodegeneration. Hence, pharmacologic modulation of GPR39 could offer an effective treatment against AD and ADRD. With the right radiotracer, PET could be used to quantify GPR39 in brains in vivo and track distribution, target engagement, and dose occupancy of novel GPR39‐based agonistic drugs to regulate Zn^2+^ (dys)homeostasis function. In this abstract we report the development and validation of the first PET radiotracer, [^11^C]TMN‐1324 to image GPR39 in rodent models of AD.

**Method:**

GPR39 agonist TM‐N1324, was selected as the lead compound for its brain penetration and high GPR39 affinity—acts with and without Zn^2+^ to both human and rodent GPR39s. To validate [^11^C]TM‐N1324 imaging potential, we performed 0‐60 min dynamic PET imaging and post‐PET biodistribution with [^11^C]TM‐N1324 in (a) normal C57BL/J mice (4 mo, n=4) and (b) male Aβ‐overexpressing APP/PS1 transgenic (TG) mice and age‐matched wild‐type (WT) mice (12 mo, n=4/group). Brain autoradiography investigated regional uptake in the same cohort. TM‐N1324 blocking studies (n=4) confirmed in vivo GPR39 specificity of the radioligand

**Result:**

Radiochemistry was fully optimized for high radiochemical purity (>98%) and molar activity (>48635 MBq/µmol) decay corrected to end‐of‐synthesis. PET imaging of rodents showed brain penetration and ∼65±4% blocking with TM‐N1324 pretreatment. It demonstrated ∼22±3% lower brain uptake in APP/PS1 TG mice compared to WT littermates (Figure 1). Autoradiography showed significant uptake in cortical regions (high GPR39‐expressing regions). Biodistribution showed ∼33±1% lower brain uptake in TG mice than WTs.

**Conclusion:**

Our pilot study here with the first GPR39‐imaging PET radiotracer, [^11^C]TM‐N1324 demonstrated brain penetration, differential uptake in mouse model of AD, and regional specificity. By validating our highly specific GPR39 PET radiotracer for studying Zn^2+^‐based dyshomeostasis, we will elucidate GPR39 PET imaging strategies to monitor target responses to potential novel therapeutics.